# Isokinetic dynamometry assessment of the transition period between eccentric and concentric hamstring contractions in athletes with a history of hamstring injury

**DOI:** 10.3389/fresc.2026.1821532

**Published:** 2026-04-29

**Authors:** Barbier Vincent, Francois Perla, Jeremy Neuillet, Aude Page, Marina Caquant, Alice Schiller

**Affiliations:** Department of Physical Medicine and Rehabilitation Unit, 3 Valleys Rehabilitation Center, Corbie, France

**Keywords:** hamstring injury, isokinetic dynamometry, plyometric test, rehabilitation, transition period

## Abstract

**Background:**

Hamstring (Hs) injuries are frequent in sports involving explosive efforts. While eccentric deficits and strength imbalances are known risk factors, the role of the transition period between eccentric and concentric contractions remains unexplored.

**Objective:**

To determine whether the transition period between eccentric and concentric contractions of the hamstrings, measured using an isokinetic dynamometer, differs between athletes with a history of Hs injury and healthy controls.

**Methods:**

This observational retrospective study included 57 participants (26 previously injured athletes and 31 healthy controls) assessed with standard and plyometric isokinetic tests. The primary outcome was the transition period between eccentric (30°/s) and concentric (240°/s) Hs contractions. Secondary outcomes included torque measurements and calculated ratios (Croisier and Plyometric).

**Results:**

The transition period did not differ significantly between groups. Injured athletes demonstrated higher concentric Hs peak torque at 240°/s (*p* = 0.002) and 60°/s (*p* = 0.013), but similar eccentric torque. No differences were found between injured and uninjured limbs in the injured group. The transition period showed no correlation with other isokinetic or anthropometric parameters.

**Conclusion:**

The transition period, as measured by isokinetic testing, does not appear to be influenced by a prior Hs injury and may lack clinical utility in guiding rehabilitation. However, plyometric evaluation may help reveal residual eccentric deficits in previously injured athletes. Future studies using electrophysiological techniques could provide more precise insight into neuromuscular latency during explosive efforts.

Levels of evidence: 3b

## Introduction

Hamstring (Hs) injury is common in sport, accounting for a quarter of muscular injuries in athletes who perform at high speeds involving explosive efforts, including sprinters and jumpers (1). Although several risk factors, such as previous Hs injury, age and agonist/antagonist ratio (2) are well documented in the literature, the end of the oscillating phase (or critical zone) appears to be a particularly vulnerable time for the onset of muscular injury to the Hs in athletes (3). In fact, this is the moment when the Hs are contracting eccentrically and the muscle fibers are under the most stress before suddenly switching to concentric contraction.

The isokinetic dynamometer enables *in vivo* muscular exercise to be studied using self-adapting resistance, at a constant speed, over a given joint amplitude. This technology provides a reliable, objective, qualitative, quantitative, reproducible and dynamic measurement of muscle strength (4). Conventional protocols for isokinetic knee tests measure maximum muscle contraction of the Hs and quadriceps (Q) in concentric mode (slow and fast speeds) and eccentric mode at slow speed (5, 6). These protocols make it possible to define a comparative right/left ratio for a muscle group, the concentric agonist/antagonist ratio for the same lower limb and the mixed Croisier ratio (7). Together, these data reflect muscular balance and have a predictive value for the risk of injury to the Hs, particularly when there is an imbalance in the Croisier ratio at the expense of the Hs (7).

The notion of relative weakness of the Hs in eccentric contraction, as well as the Q/Hs force imbalance, has led to the introduction of preventive eccentric muscle strengthening protocols in athletes to reduce the risk of Hs injuries occurring during the sporting season. Indeed, several studies using an eccentric Hs strengthening protocol have demonstrated a 60%–70% reduction in the recurrence of Hs injuries (8–13). However, there are still a significant number of athletes who experience Hs injuries after these strengthening protocols, suggesting the involvement of other factors.

We hypothesized that the usual assessment aimed at quantifying the imbalance between the quadriceps and Hs did not fully explore the sequence between the modes of contraction of the Hs. On an isokinetic dynamometer, it is possible to approximate a movement that comes close to the end of the oscillating phase during running. Literally, this involves performing a succession of eccentric (muscle-lengthening) efforts at 30°/s and concentric (muscle -shortening) efforts at 240°/s, targeting only the Hs, and determining the transition period (time period) between the two contractions.

We set up a study to determine whether the transition period between an eccentric and concentric contraction of the Hs during an “explosive” effort was different between athletes with a previous Hs injury and healthy controls. Our secondary objectives were to determine whether the transition period differed between the injured lower limb and the healthy side in injured athletes, whether there were any isokinetic parameters that affected the transition period, and finally the role of implementing routine practice with a plyometric test.

It was hoped that the information obtained from the study would be useful in guiding the resumption of activities following Hs injury and in optimizing rehabilitation protocols.

### Methods study design

This was an observational, retrospective study carried out at the 3 Valleys Rehabilitation Center (Victor Pauchet Group) in Corbie-France. Classic isokinetic tests and a new plyometric test were performed, using an isokinetic dynamometer, in athletes with a previous Hs injury and in healthy controls. Measures of muscle function (peak torque at 240°/s and 60°/s during concentric contraction of Hs and Q, peak torque at 30°/s during eccentric Hs contraction followed by peak torque at 240°/s during concentric Hs contraction, transition period, Croisier Ratio and Pliometric Ratio were compared between the two groups.

The Croisier ratio is used to assess muscle balance around the knee joint. It represents a functional ratio between antagonist and agonist muscle groups (hamstrings/quadriceps).

It is defined as the ratio of eccentric hamstring strength at 30°/s to concentric quadriceps strength at 240°/s, and is commonly used to estimate injury risk. A value between 0.8 and 1.0 is generally considered satisfactory. Lower values may reflect an eccentric hamstring deficit and have been associated with an increased risk of injury.

The plyometric ratio was developed to assess hamstring muscle function across contraction modes, by comparing fast concentric strength (240°/s) to slow eccentric strength (30°/s). This ratio is intended to better reflect the neuromuscular demands of explosive movements and to provide a clinically relevant protective reference value.

### Participants

A total of 57 participants (26 injured athletes and 31 healthy controls) were included in the study. Their demographic characteristics are presented in Table 1. Injured athletes were defined as those who practiced regular sport and had experienced a unilateral hamstring injury within the previous 3–12 months, diagnosed according to clinical criteria and ultrasound. Healthy (injury-free) controls were staff at the 3 Valleys Rehabilitation Center. Participants were eligible for inclusion if they were >15 years old and had undergone an isokinetic assessment at the 3 Valleys Rehabilitation Center between 2015 and 2023, to evaluate knee flexion and extension in the seated position. Tests were performed as part of standard practice to assess post-injury muscle recovery with a view to guiding rehabilitation management. Patients who had suffered a muscular injury <3 months previously, a painful injury (Numerical Rating Scale >4), injury with restriction of knee joint amplitudes (flexum >10°, flexion <90°), or who had undergone surgery on the injured lower limb <6 months previously, were excluded from analysis.

**Table 1:** Participant characteristics.

Demographic characteristics	Healthy *(n* *=* *31)*	Injured *(n* *=* *26)*	*P*-value
**Age (years)**	30.10 (*5.11*)	25.04 (*8.66*)	<0.001**
			
**Weight (kg)**	68.25 (*12.09*)	74.06 (*15.86*)	0.200
**Height (cm)**	167.70 (*32.10*)	174.30 (*8.92*)	0.547
**BMI (kg/m^2^)**	22.49 (*2.23*)	24.25 (*4.10*)	0.069
**Sex (F/M), n**	15/16	12/14	0.273
**Sport intensity, n**			0.001**
***Amateur***	31	16	
***High intensity***	0	10	
***Professional***	0	0	
**Total Hs injuries both sides, n**			<0.001**
** *0***	27	0	
** *1***	3	10	
** *2***	1	5	
** *3***	0	6	
** *4***	0	1	
** *5***	0	1	
** *>5***	0	3	
**Contralateral Hs injury, *n***			0.008*
** *0***	28	17	
** *1***	3	5	
** *2***	0	2	
** *Multiple***	0	1	
** *Unknown***	0	1	
**Other muscle injuries, %.**			**0.063**
** *0***	30	21	
** *1***	1	4	
** *Unknown***	0	1	

Data are means (standard deviation) for age, weight, height and BMI. BMI, body mass index; F, female; M, male; n, number; Hs, hamstring. Statistically significant difference * *p* ≤ 0.01, ** *p* ≤ 0.001.

Control data were collected using a series of tests developed on healthy subjects for the purpose of simulating the use of machines regularly updated by the establishment.

Healthy controls were slightly older than injured athletes, whereas weight, height, BMI and gender distribution were comparable between the two groups (Table 1). Injured athletes had a higher frequency of intense sport practice than controls, as well as a greater history of hamstring injuries and a higher rate of contralateral Hs injuries. However, there were no group differences in global muscular wounds.

This study was declared to the National Commission for Freedom of Information Technology act and was approved by the Adene Ethics Committee on 05/09/2024 (reference number: IRB_ADENE_20240901). All participants included in this study were sent an information letter including non-objection to the use of their data for research purposes, from the clinical research unit of the Victor Pauchet clinic. The absence of an unfavorable response within 15 working days was considered as a non-objection.

### Procedures demographic measures

All demographic data, including sex, age, height, weight, BMI, intensity of sport practiced, number of Hs lesions on both sides, history of Hs lesions on the contralateral side and other muscle injuries, were collected using the study questionnaire completed by the doctor during the participant's visit (Supplementary Appendix 1).

### Muscle function measures

All measures were carried out using a CON-TREX Multi-Joint system isokinetic dynamometer with adapter ref. #5001 and stabilizer ref. #5007. This was used to evaluate knee flexion and extension in the seated position at a frequency set at 256 Hz. Firstly, using the classic isokinetic test, data were collected during a concentric contraction of the quadriceps (Q) followed by a concentric contraction of the hamstrings (Hs) at fast speed (240°/s) and then at slow speed (60°/s). Secondly, for the plyometric evaluation, a slow eccentric Hs contraction test at 30°/s was performed, followed by a fast concentric Hs contraction at 240°/s. The set-up and testing procedures were identical for all participants, as defined by the machine's standardized parameters and commonly accepted practice. The absolute value of the transition period was calculated using the raw data from the machine, after converting this data in excel and counting the number of sequences of absence of movement between an eccentric and concentric contraction. The recording frequency of the isokinetic dynamometer was 256 Hz, so that each recording was spaced at 3.9 ms apart. We therefore multiplied the number of recordings by 3.9 to determine the transition period.

### Statistical analysis

All statistical analyses were carried out using SPSS statistics˝ v25 software. Qualitative data are presented as numbers (n). Quantitative data are presented as means and standard deviations (m, (SD).

## Results

Muscle function measures in injured athletes and healthy controls are presented in Table 2. During the classic isokinetic test, Hs peak torque at 240°/s (*p* = 0.001) and Hs peak torque at 60°/s (*p* = 0.013) were higher in injured athletes than in healthy controls. There were no significant group differences in Q peak torque or in the Croisier ratio.

**Table 2:** Muscle function measures: healthy controls vs. injured athletes.

Test	Healthy (*n* = 31)	Injured (*n* = 26)	*P*-value
***Classic isokinetic test***			
** *PT Q 240°/s concentric (Nm/kg)***	1.77 (0.36)	1.91 (0.32)	0.068
** *PT Hs 240°/s concentric (Nm/kg)***	0.95 (0.21)	1.29 (0.47)	**0**.001**
** *PT Q 60°/s concentric (Nm/kg)***	2.56 (0.53)	2.66 (0.50)	0.564
** *PT Hs 60°/s concentric (Nm/kg)***	1.33 (0.30)	1.64 (0.53)	**0**.013*
***Plyometric test***			
** *PT Hs 30°/s eccentric (Nm/kg)***	1.67 (0.39)	1.78 (0.41)	0.301
** *PT Hs 240°/s concentric (Nm/kg)***	0.88 (0.24)	1.18 (0.44)	**0**.002*
** *Croisier ratio***	0.96 (0.20)	0.95 (0.23)	0.701
** *Plyometric ratio***	0.56 (0.22)	0.70 (0.21)	**0**.005*
** *Transition period (ms)***	99.20 (5.34)	104.77 (21.99)	0.273

Data are means (standard deviation), n, number. Statistically significant difference * *p* ≤ 0.05, ** *p* ≤ 0.001. PT, peak torque; Q, quadriceps; Hs, hamstring.

During the plyometric test, slow-speed Hs peak torque at 30°/s was similar but high-speed Hs peak torque at 240°/s was higher in injured athletes compared to healthy controls (*p* = 0.002). Consequently, the calculated plyometric ratio was also significantly higher in the injured group (*p* = 0.005). Nevertheless, there was no significant difference in the transition period between the two groups.

Muscle function measures in injured athletes (*n* = 26), comparing healthy vs. injured lower limbs, are presented in Table 3. There were no significant differences in either classic isokinetic or plyometric measures, between the healthy and injured limbs of athletes. The transition period for healthy and injured limbs was comparable and was unrelated to any other isokinetic measure or anthropometric data (Supplementary Appendix 2).

**Table 3:** Muscle function measures in injured athletes: healthy vs. injured lower limb.

Test	Injured Side	Healthy Side	*P*-value
***Classic isokinetic test***			
** PT Q 240°/s, concentric**	1.85 (*0.31*)	1.97 (*0.36*)	0.146
** PT Hs 240°/s, concentric**	1.26 (*0.49*)	1.31 (*0.48*)	0.534
** PT Q 60°/s, concentric**	2.68 (*0.47*)	2.65 (*0.49*)	0.756
** PT Hs 60°/s, concentric**	1.59 (*0.54*)	1.68 (*0.56*)	0.31
** *Plyometric test***			
** PT Hs 30°/s, eccentric**	1.69 (*0.43*)	1.87 (*0.42*)	0.156
** PT Hs 240°/s, concentric**	1.20 (*0.69*)	1.16 (*0.29*)	0.41
** Croisier ratio**	0.97 (*0.26*)	0.92 (*0.22*)	0.558
** Plyometric ratio**	0.72 (*0.33*)	0.67 (*0.21*)	0.978
** Transition period (ms)**	106.79 (*28.97*)	102.75 (*15.86*)	0.729

Data are means (standard deviation), *n* = 26 for all measures. PT, peak torque; Q, quadriceps; Hs, hamstring.

## Discussion

This study set out to investigate whether the transition period between an eccentric and a concentric Hs muscle contraction during an explosive effort, differed between athletes with a previous Hs injury and healthy controls.

The transition period is essential, particularly during rapid, explosive movements of the lower limbs, where a short delay between these two modes of contraction (stretching-shortening cycle) optimizes performance (14). To the best of our knowledge, this is the first study to investigate the transition period using data from an isokinetic dynamometer. Using this device, we set up a plyometric test including a slow-speed eccentric (30°/s) sequence followed by a fast-speed concentric (240°/s) effort, in order to approximate an explosive effort *in vivo*.

The results of our study suggest that the transition period, calculated in this way, is not influenced by Hs injury. Compared to healthy controls, athletes with a Hs injury in the previous 3–12 months, had a higher Hs concentric peak torque at 240°/s and 60°/s during the classic isokinetic test, and a higher Hs concentric peak torque at 240°/s during plyometric testing. This may be due to the fact that 10/26 injured athletes but 0/31 controls practiced high-intensity sports. Nevertheless, we found no significant difference in the transition period between injured athletes and healthy controls. This suggests that, irrespective of the intensity of sporting activity, and despite higher torque peaks, the transition period was not affected by previous Hs injury. Furthermore, no relationship was found between the transition period and any other clinical or isokinetic parameters.

As the transition period was similar for each analysis, we concluded that either it is not a useful isokinetic parameter for guiding the recovery of athletes with Hs injuries, or the isokinetic dynamometer is not the most accurate measurement tool for this parameter. Increasing the isokinetic dynamometer frequency could generate a greater number of sequence recordings, thereby increasing the accuracy of measurements. Thus, we cannot state with certainty that the transition period was not modified, given the potential lack of measurement precision. Although it is technically feasible to measure transition period using an isokinetic dynamometer, practical constraints seem to limit its routine use in the field of Hs injuries.

Future investigations could compare isokinetic data with electrophysiological data, which would be more accurate. Given the unexplained residual recurrence of lesions in the Hs (9), an electrophysiological approach to transition time may help to better understand the different isokinetic parameters guiding rehabilitation.

A secondary aim of our study was to test the relevance of a plyometric test in routine practice, including a slow-speed sequence followed by a fast-speed effort, in order to approximate an explosive effort *in vivo*.

In injured athletes, compared to healthy controls, Hs eccentric peak torque at 30°/S was similar while Hs concentric peak torque at 240°/s was higher in plyometric testing. This suggests that the eccentric contraction is deficient at slow speed in athletes with a history of Hs injury (15). Nevertheless, despite this deficit, they are able to develop more muscle strength in a plyometric sequence than a less athletic individual. The plyometric test may be useful in detecting a possible eccentric deficit during explosive effort, by completing the isolated measurement of the Hs eccentric peak torque in the classic test.

This study found no difference in any of the classic isokinetic or plyometric measures, including transition period, between healthy and injured limbs of athletes. We cannot rule out the possibility that athletes with an injury history of 3–12 months had fully recovered. The mean time between injury and testing was 9.2 months, and the average recovery time for a muscle injury is around 6 weeks. Ideally, a similar study should be carried out, comparing individuals practicing a similar physical activity, divided into one group with a Hs injury and one without.

These results should be interpreted with caution. Notably, nearly one third of the injured athletes were engaged in high-intensity sports, which may have influenced muscle performance. In this context, higher peak torque during slow-velocity eccentric contractions might have been expected in this group.

To draw more definitive conclusions, it would be necessary to ensure comparable levels of sports participation between injured and healthy subjects. Nevertheless, these findings highlight the need for further investigation into peak torque during slow eccentric contractions.

In addition, the relatively small sample size limits the statistical power of the analysis.

## Conclusion

The findings of this exploratory study suggest that the transition period, calculated using data from an isokinetic dynamometer, is not affected by a history of Hs injury. Nevertheless, plyometric evaluation of athletes performing explosive efforts could be useful for understanding the different risk factors involved in Hs injury. In our study, an eccentric deficit seemed to persist in injured athletes despite rehabilitation during plyometric effort. Future research should investigate the transition period during an explosive effort using electrophysiological recording of neuromuscular latency to increase measurement precision.

## Data Availability

The raw data supporting the conclusions of this article will be made available by the authors, without undue reservation.
