# Supplementary material for: Isokinetic dynamometry assessment of the transition period between eccentric and concentric hamstring contractions in athletes with a history of hamstring injury
Source: Front Rehabil Sci. 2026 Apr 29;7:1821532. doi: 10.3389/fresc.2026.1821532 (PMC13168000; doi:10.3389/fresc.2026.1821532)
Supplement: Supplementary file 1 [file Supplementaryfile1.pdf]

Appendix 1 QUESTIONNAIRE

HAMSTRING PLYOMETRIC TEST ON ISOKINETIC DYNAMOMETER

DATE: ....

NAME:...

FIRST NAME: ....

DATE OF BIRTH: .... **SEX:** F !" M !"

INJURED SIDE: Right !" Left !"

DOMINANT LEG: Right !" Left !" Unknown !"

HEIGHT: .... **WEIGHT:** .... **BMI:** ....

MEDICAL HISTORY:

- Total number of injuries to the injured hamstring side: _
- Number of injuries to the injured side in the last 12 months: ___
- Date of last injury to the injured hamstring: ___ / ____ / _____
- Contralateral hamstring injury: Yes !" No !"
- **Other injuries:**
    - Lower limb muscle injuries: Yes !" No !" → Which? : ....
    - Ankle sprain: Yes !" No !" → Which side and when?: ....
    - Surgery of lower limbs: Yes !" No !" → Side, type, and date: .... o Cardiopulmonary issues: ....

PAIN:

- Spontaneous pain (VAS): ____ /10
- **Location:** ....
- Normal training possible without pain: Yes !" No !"
- Training with <80% intensity possible: Yes !" No !"
- Normal training possible: Yes !" No !"

- Sprinting possible: Yes !" No !"
- Running between 60% and 80% possible: Yes !" No !"
- Running <50% of maximum possible: Yes !" No !"
- Pain while walking: Yes !" No !"

!

PHYSICAL EXAMINATION:

- Knee joint limitation: Yes !" No !"
- Contralateral knee joint limitation: Yes !" No !"
- Swelling (edema): Yes !" No !"
- **Muscle strength:**
  - Flexion: /5 Extension: /5 Inversion: /5 Eversion: /5
- **Thigh circumference at 10 cm from patella:** - Right thigh: ....
  - Left thigh: ....

TREATMENT:

- Eccentric rehabilitation after injury: Yes !" No !"
- Ongoing rehabilitation: Yes !" No !"
- Previous test on isokinetic dynamometer: Yes !" No !"
  → If yes, specify results: ....
